# FA-97, a New Synthetic Caffeic Acid Phenethyl Ester Derivative, Protects against Oxidative Stress-Mediated Neuronal Cell Apoptosis and Scopolamine-Induced Cognitive Impairment by Activating Nrf2/HO-1 Signaling

**DOI:** 10.1155/2019/8239642

**Published:** 2019-12-03

**Authors:** Ting Wan, Zihao Wang, Yi Luo, Yifan Zhang, Wei He, Yu Mei, Jincheng Xue, Min Li, Huafeng Pan, Weirong Li, Qi Wang, Yujie Huang

**Affiliations:** ^1^Science and Technology Innovation Center, Guangzhou University of Chinese Medicine, Guangzhou 510405, China; ^2^Institute of Clinical Pharmacology, Guangzhou University of Chinese Medicine, Guangzhou, Guangdong 510006, China; ^3^Institute of Brain and Gut Axis (IBAG), Centre of Clinical Research for Chinese Medicine, School of Chinese Medicine, Hong Kong Baptist University, Kowloon Tong, Hong Kong SAR, China; ^4^Department of Chemistry, Southern University of Science and Technology, Shenzhen, Guangdong 518055, China; ^5^Clinical Medical College of Acupuncture Moxibustion and Rehabilitation, Guangzhou University of Chinese Medicine, Guangzhou, Guangdong 510006, China

## Abstract

Alzheimer's disease (AD) is an age-related neurodegenerative disorder with cognitive deficits, which is becoming markedly more common in the world. Currently, the exact cause of AD is still unclear, and no curative therapy is available for preventing or mitigating the disease progression. Caffeic acid phenethyl ester (CAPE), a natural phenolic compound derived from honeybee hive propolis, has been reported as a potential therapeutic agent against AD, while its application is limited due to the low water solubility and poor bioavailability. Here, caffeic acid phenethyl ester 4-*O*-glucoside (FA-97) is synthesized. We validate that FA-97 attenuates H_2_O_2_-induced apoptosis in SH-SY5Y and PC12 cells and suppresses H_2_O_2_-induced oxidative stress by inhibiting the ROS level, malondialdehyde (MDA) level, and protein carbonylation level, as well as induces cellular glutathione (GSH) and superoxide dismutase (SOD). Mechanistically, FA-97 promotes the nuclear translocation and transcriptional activity of Nrf2 associated with the upregulated expression of HO-1 and NQO-1. The prime importance of Nrf2 activation in the neuroprotective and antioxidant effects of FA-97 is verified by Nrf2 siRNA transfection. In addition, FA-97 prevents scopolamine- (SCOP-) induced learning and memory impairments *in vivo* via reducing neuronal apoptosis and protecting against cholinergic system dysfunction in the hippocampus and cortex. Moreover, the increased MDA level and low total antioxidant capacity in SCOP-treated mouse brains are reversed by FA-97, with the increased expression of HO-1, NQO-1, and nuclear Nrf2. In conclusion, FA-97 protects against oxidative stress-mediated neuronal cell apoptosis and SCOP-induced cognitive impairment by activating Nrf2/HO-1 signaling, which might be developed as a therapeutic drug for AD.

## 1. Introduction

Alzheimer's disease (AD) is a progressive neurodegenerative disorder and a leading cause of cognitive deficits, memory loss, and behavioral alterations in an aging population worldwide [1]. Currently, AD accounts for 50 million cases in the world, and this number will be more than triple to 152 million by 2050 [2]. The pathological hallmarks of AD are amyloid deposition, tau protein hyperphosphorylation and accumulation, neuronal dystrophy, oxidative stress and decline in acetylcholine (ACh) levels, etc. [3]. However, the exact pathogenesis of AD is still unclear, and no curative therapy is available for the prevention or mitigation of the disease progression till date. Current treatment strategies encompass the use of FDA-approved medications like acetylcholinesterase inhibitors (AChEIs) and N-methyl-D-aspartate (NMDA) receptor antagonist [4], which help to mask behavioral changes and some of the effects of memory deficiency, while not treating the disease itself [5]. It is as urgent as ever for researchers to develop innovative treatment strategies to fight this disease.

Oxidative stress results from an imbalance between the formation of free radicals and the impaired ability of organisms to detoxify these reactive intermediates or to repair the damage that they cause [6]. Free radicals are generally known as reactive nitrogen species (RNS) or reactive oxygen species (ROS), such as the hydroxyl radical (^·^OH), the superoxide radical anion (O_2_^·^¯), and hydrogen peroxide (H_2_O_2_) [7]. Under physiological conditions, small amounts of ROS do not cause damage but coordinate with the body's antioxidant system to maintain homeostasis, involving a balance between prooxidants and antioxidants comprised of low molecular weight antioxidant species (e.g., vitamins E and C and carotenoids) and larger molecular weight antioxidant enzymes, such as superoxide dismutase (SOD), catalase (CAT), glutathione peroxidase (GPx), and the thioredoxin (TRX) system [8]. However, once ROS overwhelms the cellular antioxidant activity, oxidative stress occurs, leading to the accumulation of cytotoxic compounds that result in not only protein collapse, enzyme failure, and lipid destruction but also destruction of the majority of neurons, which plays an important role in the pathogenesis of AD [9, 10]. Recent experiments have confirmed the plausible mechanism of antioxidant therapeutics in AD by free radical scavenging activity, leading to inhibition of hydrogen superoxide and thereby inhibiting amyloid deposition in neuronal cells [3, 11]. Antioxidative options, including some new neuroprotective agents that eliminate excess reactive oxygen species efficiently, have a certain therapeutic effect on AD [3, 12].

The nuclear factor erythroid 2- (NF-E2-) related factor 2 (Nrf2), a basic region-leucine zipper transcription factor, maintains cellar redox homeostasis by regulating the expression of various antioxidant proteins [13, 14]. Under homeostatic conditions, Nrf2 is sequestered by the E3 ligase adapter Kelch-like ECH-associated protein 1 (Keap1) in the cytoplasm and is hence presented to degradation through the ubiquitin proteasome system [14]. Upon exposure to oxidative stress, Nrf2 escapes from Keap1-mediated degradation by dissociating from the Nrf2-Keap1 heterodimer and then translocates into nuclear to recognize an enhancer sequence-termed antioxidant response element (ARE), which encodes a network of cooperating enzymes involved in antioxidant metabolism including hemeoxygenase-1 (HO-1), GPx, and quinone oxidoreductase-1 (NQO-1) [15]. It has been reported that AD patients show reduced nuclear levels of Nrf2 in hippocampal neurons [16, 17]; NQO-1, HO-1, SOD1, glutathione synthetic enzymes, and Nrf2 levels in hippocampal neurons are reduced in APP/PS1 transgenic AD mice and 3xTG model of AD [18, 19]; several Nrf2 inducers alleviated cognitive defects in transgenic AD animal models showing anti-AD potency [20–23]. All of these evidences highlight the protective role of Nrf2 in neurodegenerative conditions, and an emerging target against oxidative stress in AD is given by the Keap1/Nrf2/HO-1 pathway [24, 25].

**Figure 1:**
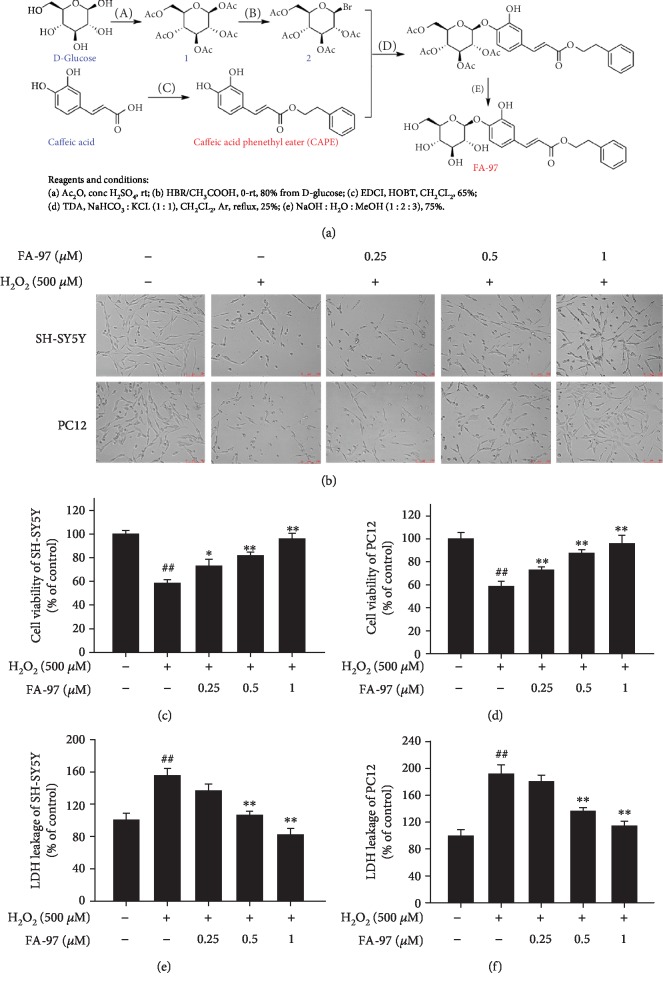
Effect of FA-97 on H_2_O_2_-induced cytotoxicity in SH-SY5Y and PC12 cells. SH-SY5Y and PC12 cells were plated in a 96-well plate, treated with H_2_O_2_ (500 *μ*M) and FA-97 (0, 0.25, and 0.5, 1 *μ*M) for 24 h. (a) Synthesis scheme of FA-97. FA-97 is synthesized via the coupling reaction between an acetyl-protected brominated D-glucose and caffeic acid phenethyl ester (CAPE) starting from commercially available caffeic acid. (b) Morphological changes in SH-SY5Y and PC12 cells were observed by phase contrast microscopy. (c, d) The viability of SH-SY5Y (c) and PC12 cells (d) was tested by CCK8 assay. (e, f) Effects of FA-97 on the released LDH of SH-SY5Y (e) and PC12 cells (f) induced by H_2_O_2_ were detected. Data from three times independent experiments were expressed as means ± SD. ^#^*P* < 0.05 and ^##^*P* < 0.01 compared with the control group and ^∗^*P* < 0.05 and ^∗∗^*P* < 0.01 compared with the H_2_O_2_-treated group.

Caffeic acid phenethyl ester (CAPE) is a natural phenolic compound occurring in a variety of plants and derived from honeybee hive propolis [26]. It has been reported that CAPE protects neuronal cells against cisplatin-induced neurotoxicity [27, 28], counteracts oxidative stress, and decreases neuronal apoptosis and neuroinflammation, as well as improves learning and memory ability in AD mice [29] with no side effects, which could be a potential therapeutic agent as a neuroprotective agent against progressive AD [30, 31]. However, the CAPE molecule is unstable for decomposing easily in biological systems due to its ester bond (*α*-*β* unsaturated carbonyl) and the catechol groups (Figure 1(a)) [32]. Moreover, the application of CAPE *in vivo* is also limited due to its low water solubility and poor bioavailability [33, 34].

In this study, to overcome the shortcomings of CAPE, FA-97 (caffeic acid phenethyl ester 4-*O*-glucoside) was synthesized via the coupling reaction between an acetyl-protected brominated D-glucose and CAPE starting from commercially available caffeic acid (Figure 1(a)). This synthetic process has good yields and FA-97 has better water solubility than CAPE. Moreover, FA-97 was found to protect against oxidative stress-mediated apoptosis of neuronal cells *in vitro* and ameliorate scopolamine-induced cognitive impairment *in vivo*. Further mechanistic studies revealed that Nrf2 activation was involved in the neuroprotective effect of FA-97 by suppressing oxidative stress *in vitro* and *in vivo*.

## 2. Materials and Methods

### 2.1. Reagents and Antibodies

FA-97 (caffeic acid phenethyl ester 4-*O*-glucoside, C_23_H_26_O_9_, MW = 446.16 g/mol) (>99% purity) is synthesized via the coupling reaction between an acetyl-protected brominated D-glucose and caffeic acid phenethyl ester (CAPE) starting from commercially available caffeic acid. D-glucose was dissolved in anhydrous Ac_2_O and concentrated H_2_SO_4_ was added at 0°C. Then, the solution was allowed to room temperature and stirred overnight. Water and EtOAc were added at 0°C, and the resulting mixture was then extracted with EtOAc. The combined organic layers were dried over Na_2_SO_4_ and concentrated under reduced pressure. FA-97 was dissolved in dimethyl sulfoxide (DMSO) as stock solution at 0.1 M and stored at -20°C. CAPE (cat #C8221) and D-glucose (cat #158968) were purchased from Sigma-Aldrich (St. Louis, MO, USA). Scopolamine (SCOP, cat #D-066) was purchased from Chengdu Herbpurify Co., Ltd. (Chengdu, China). Donepezil (DNP, cat #110119-84-1) was obtained from Yuanye Biological Co., Ltd. (Shanghai, China). N′,N-Dimethylacetamide (DMAC) (cat #NO. A504006) was purchased from Sangon Biotech (Shanghai, China), and polyoxyl 15 hydroxystearate (cat #MB1809) was obtained from Dalian Meilun Biotechnology Co., Ltd. (Dalian, China). DMSO and hydrogen peroxide (H_2_O_2_) were obtained from Sigma Chemical Co., Ltd. (St. Louis, MO). Dye 4,6-diamidino-2-phenylindole (DAPI) was obtained from Roche Diagnosis Co., Ltd. (Shanghai, China).

Primary antibodies against *β*-actin (cat #3700), Bcl-2 (cat #3498S), Bax (cat #2772S), Cytochrome c (cat #11940S), Caspase-9 (cat #9508S), hemeoxygenase-1 (HO-1) (cat #70081S), and Lamin A/C (cat #4777) were purchased from Cell Signaling Technology (Danvers, MA). Primary antibody against Nrf2 (cat #ab62352), GAPDH (cat #ab8245), and Lamin C (cat #ab125679) were obtained from Abcam, Inc. (Cambridge, UK). Primary antibody against NQO-1 (cat #abs115592) was purchased from Absin Bioscience Inc. (MD, USA).

### 2.2. Cell Culture and Treatment

SH-SY5Y and PC12 cell lines were purchased from the Cell Bank of Shanghai Institute of Biochemistry and Cell Biology at the Chinese Academy of Sciences (Shanghai, China). SH-SY5Y cells were cultured in Dulbecco's modified Eagle's medium (DMEM), and PC12 cells were cultured with RPMI-1640 medium supplemented with 10% (*v*/*v*) fetal bovine serum (FBS), penicillin (100 U/ml), and streptomycin (100 *μ*g/ml) (Gibco BRL, Gaithersburg, MD, USA). Cells were maintained in a stable environment with 5% CO_2_ at 37°C. For drug administration, cells were treated with different concentrations of FA-97 (0.25, 0.5, 1 *μ*M) and H_2_O_2_ (500 *μ*M) for 24 h. The FA-97 stock solution was freshly diluted with culture medium to the final concentration, and the final DMSO concentration did not exceed 0.1% with no effect on cell viability.

### 2.3. Animals

Male Kunming (KM) mice (18-22 g) were supplied by the Experimental Animal Center of Guangzhou University of Chinese Medicine (Guangzhou, China). All animals were maintained at 23 ± 2°C, with a 12 h light/dark cycle and a relative humidity 45 ± 10%, with free drinking and eating. All experimental procedures were in accordance with the National Institute of Health Guide for the Care and Use of Laboratory Animals (Bethesda, MD, USA) and were carried out under the approval of the animal ethics Committee of Guangzhou University of Chinese Medicine.

After acclimatization for 7 days, mice were randomly assigned to seven groups: control group, scopolamine- (SCOP-) treated (3 mg/kg) group, scopolamine+FA-97-treated (2.5 mg/kg) group, scopolamine+FA-97-treated (5 mg/kg) group, scopolamine+FA-97-treated (10 mg/kg) group, scopolamine+CAPE-treated (10 mg/kg) group, and scopolamine+donepezil- (DNP-) treated (3 mg/kg) group (*n* = 12). FA-97 and CAPE were prepared daily with saline solution containing 5% (*v*/*v*) N′,N-dimethylacetamide and 5% (*v*/*v*) polyoxyl 15 hydroxystearate as intragastric administration. FA-97, CAPE, and DNP treatments were given by oral gavage once per day for 30 days. Mice were administrated intraperitoneally with SCOP (3 mg/kg) from the 21th days, while mice in the control group were administrated intraperitoneally with saline. All mice underwent behavior tests 30 min after SCOP injection ([Supplementary-material supplementary-material-1]). After finishing all behavior tests, mice were sacrificed for sample collection on the 30th day. Eight mice in each group were randomly sacrificed by cervical dislocation to remove brains rapidly, which were cleaned with phosphate buffer (PBS, 0.1 M, pH = 7.4) on ice, and then the hippocampus and cortex were carefully dissected and stored at -80°C for further analysis. The other four mice were anesthetized with chloral hydrate (10%) and perfused through the left ventricle with normal saline, following by paraformaldehyde (4%). After the perfusion, brains were removed and submerged in paraformaldehyde (4%) for further pathological and immunohistochemical studies.

### 2.4. Cell Viability Assay

The CCK8 assay was used to evaluate the effect of FA-97 on the viability of SH-SY5Y and PC12 cells. Cells were plated into 96-well plates at a density of 2 × 10^5^ cells/well in medium and cultured overnight. In the preliminary experiment, SH-SY5Y and PC12 cells were treated with H_2_O_2_ (0, 25, 50, 100, 200, 300, 400, 500, and 600 *μ*M) or FA-97 (0, 0.125, 0.25, 0.5, 1, 2, and 3 *μ*M), respectively. For formal experiments, cells were treated with H_2_O_2_ (500 *μ*M) and different concentrations of FA-97 (0, 0.25, 0.5, and 1 *μ*M). After 24 h, 20 *μ*l CCK8 solution (cat #A311-01/02, Vazyme Biotech Co., Ltd., Nanjing, China) was added into the medium and incubated for 45 min. The absorbance was measured at 450 nm.

### 2.5. Lactate Dehydrogenase (LDH) Release Assay

The LDH released from SH-SY5Y and PC12 cells was determined by commercial LDH assay kit (cat #KGT02448) from KeyGen BioTech (Nanjing, China). Briefly, 100 *μ*l cell culture medium was harvested and mixed with buffer A (250 *μ*l) and buffer B (50 *μ*l). After 37°C water bath for 15 min, buffer C (250 *μ*l) was added and incubated at 37°C for another 15 min. The absorbance was measured at 440 nm.

### 2.6. Annexin V/PI Staining

Apoptosis-mediated cell death of nerve cells was examined using a FITC-labeled Annexin V/PI Apoptosis Detection Kit (KeyGen Biotech, Nanjing, China) according to the manufacturer's instructions. After being harvested and washed with PBS twice, SH-SY5Y and PC12 cells were resuspended in 500 ml binding buffer, followed by adding Annexin V-fluorescein isothiocyanate (5 *μ*l) and PI (5 *μ*l). Then, cells were incubated in the dark for 30 min at room temperature. Flow cytometry (Beckman Coulter, Inc., USA) analysis was done immediately after supravital staining.

### 2.7. Western Blot Analysis

The cell extracts of SH-SY5Y or PC12 were obtained by lysis with RIPA buffer. For brain samples, the hippocampus and cortex were homogenized in ice-cold RIPA buffer containing PMSF (1 : 100), protease inhibitor and phosphatase cocktail (1 : 100) for 30 minutes. The lysate was centrifuged for 15 min (12000 rpm, 4°C), and the supernatant was collected as protein sample of brain. Then, the BCA assay was performed to quantify the protein concentration. Protein samples were separated by SDS-PAGE and transferred to a polyvinylidene difluoride (PVDF) membrane (Millipore, Billerica, MA). Membranes were blocked by 5% BSA for 1.5 h at room temperature, incubated with the primary antibodies specific for target proteins overnight at 4°C, and then incubated with the secondary antibody for 1 h at room temperature. Detection was performed by the Odyssey Infrared Imaging System (LI-COR Inc., USA) using a fluorescent readout and quantified using Bio-Rad Image Lab 5.2.1 software (Bio-Rad Laboratories, California, USA).

### 2.8. Reactive Oxygen Species (ROS) Assay

The assay was performed to analyze the levels of ROS in SH-SY5Y and PC12 cells by using fluorescent dye 2′7′-dichlorofluorescein-diacetate (DCFH-DA, cat #S0033, Beyotime Institute of Biotechnology, Shanghai, China). The nonfluorescent DCFH-DA can be oxidized to fluorescent 2′7′-dichlorofluorescein (DCF) by ROS. SH-SY5Y and PC12 cells on coverslips were fixed with 4% paraformaldehyde, incubated with DCFH-DA for 20 min at 37°C in the dark, washed with medium three times to remove the extra DCFH-DA, and then photographed by fluorescence microscope (Leica Microsystems, Heerbrugg, Switzerland). To quantify the ROS level, cells were collected and incubated with DCFH-DA for 30 min at 37°C in the dark and then assessed by a spectrofluorometer at an excitation wavelength of 488 nm and an emission wavelength of 525 nm. Parallel blanks were used to standardize DCF. ROS levels were quantified from a DCF standard curve.

### 2.9. Measurement of Malondialdehyde (MDA), Glutathione (GSH), Protein Carbonyl, and Superoxide Dismutase (SOD) Activity

SH-SY5Y and PC12 cells were treated with H_2_O_2_ (500 *μ*M) and FA-97 (0, 0.25, 0.5, and 1 *μ*M) for 24 h, and then the supernatant of cell homogenates was collected. Then, the level of MDA, GSH, protein carbonyl content, and SOD activity was measured according to the manufacturer's instructions of the MDA assay kit (#KGT004), GSH assay kit (#KGT006), and SOD activity assay kit (#KGT00100-1) from KeyGen BioTech (Nanjing, China) and the protein carbonyl content assay kit (#DTG-1-G) obtained from Comin Biotechnology Co., Ltd. (Suzhou, China), respectively.

### 2.10. Luciferase Reporter Assay

The transcriptional activity of Nrf2 was determined using an ARE Reporter kit (BPS Bioscience, San Diego, CA, USA). Briefly, SH-SY5Y and PC12 cells were cotransfected for 24 h with ARE-luciferase reporter plasmid and a plasmid that constitutively expressed Renilla luciferase using Lipofectamine™ 2000 (Invitrogen; Thermo Fisher Scientific, Inc.). After serum recovery, cells were treated with H_2_O_2_ (500 *μ*M) and FA-97 (0, 0.25, 0.5, and 1 *μ*M) for 24 h. The ARE-luciferase activities were determined using a luciferase assay kit in accordance with the manufacturer's instructions (Promega, Madison, WI). Data were normalized with Renilla luminescence and obtained from three independent experiments.

### 2.11. Immunofluorescence Staining

SH-SY5Y and PC12 cells were grown on coverslips and treated with H_2_O_2_ (500 *μ*M) and FA-97 (0, 0.25, 0.5, and 1 *μ*M) for 24 h. Cells were fixed with 4% paraformaldehyde, permeabilized in 0.2% Triton X-100, and incubated with 3% BSA. After being incubated with primary Nrf2 antibody, cells were exposed to a secondary antibody and stained with DAPI. Cells were observed and photographed with a confocal laser-scanning microscope (FluoView FV 1000, Olympus, Tokyo, Japan).

### 2.12. Molecular Docking Studies

Molecular docking simulations were used to explore the potential interaction of FA-97 on Nrf2. The crystal structure of Nrf2 (PDB: 6QMC) was prepared by the Protonate 3D tool in MOE (version 2010.10, Chemical Computing Group Inc. Montreal, Quebec, Canada, 2010), and all the water molecules were removed. Hydrogen atoms were added using MOE. The structure of FA-97 was modeled and minimized in MOE. Docking simulations were carried out in the CDOCKER module implemented in Discovery Studio 2.5.5 (version 2.5, Accelrys Inc., San Diego, CA, 2009).

### 2.13. Transfection of Nrf2 siRNA

Nrf2 siRNA sequence was purchased from (Thermo Fisher Scientific, Hudson, NH, United States). SH-SY5Y cells were plated in six-well plates with fresh medium. Nrf2 siRNA or nontargeting siRNA (NT siRNA) transfection was performed according to the manufacturer's instructions of Lipofectamine 2000 reagent (Invitrogen, Carlsbad, CA, USA). Cells were cultured in serum-free medium for 8 h and then treated with H_2_O_2_ (500 *μ*M) and FA-97 (0, 0.25, 0.5, and 1 *μ*M) for 24 h.

### 2.14. Morris Water Maze Test

The Morris water maze test was used to assess spatial learning and memory ability of mice after FA-97 treatment. The experiment was carried out in a round stainless steel tank (diameter: 120 cm, height: 50 cm), which was divided into four equal quadrants with a black plexiglass escape platform (diameter: 10 cm) located in the center of any quadrant. The tank was filled with water (temperature: 23 ± 2°C) to a depth of 30 cm, and the escape platform was placed 1 cm below the water surface. The first day was adaptive training day. On the later five formal experiment days, a mouse was placed at one of the starting points facing the wall and released into the pool. The escape latency was recorded from the starting point to find the hidden platform and analyzed using the record system. If the mouse failed to find the platform within 60 s, the escape latency was recorded as 60 s. Each mouse was manually guided to the platform to strengthen memory for 10 s. The procedure was repeated with each mouse starting in each of the four quadrants stochastically changed on each day. The spatial probe test was carried out on the seventh day. The underwater platform was removed, and each mouse was allowed to swim freely for 60 s. The swimming speed, time spent in the target quadrant, and the crossing times of the platform were measured to evaluate retention of spatial memory.

### 2.15. New Object Recognition Test

New object recognition experiment was carried on in a bright testing arena (length: 40 cm; width: 40 cm; height: 40 cm). Two identical objects (A1 and A2) were placed in the relative position. Mice were placed into the experimental device in a back-to-back manner. After a 5-minute exploration, mice were taken out and put back into the animal cage. 24 hours later, one of the two identical objects was replaced with another different object referred to as a novel object (B) and the mouse was put into the arena again. The time for exploring the novel object within 5 minutes was recorded. Mice were familiarized with the position of the object and the novel object in turn to reduce the error during the test period. In order to eliminate the influence of odor, objects and the experimental device should be cleaned in time.

### 2.16. Cresyl Violet (Nissl) Staining

Cresyl violet (Nissl) staining was performed for histopathological analysis to assess the degree of neuronal cell death. The brain sections were deparaffinized, rehydrated, and followed by staining with a cresyl violet (0.5%) solution (cat #C0117, Beyotime Biotechnology) for 10 min. After that, the slides were washed with distilled water twice and dehydrated in a graded ethanol series (70%, 95%, and 100%, for 1 min each), followed by immersion in xylene. Finally, the slides were covered with glass coverslips with neutral resin. The slides were then examined and analyzed by a light microscope and LEICA QWin Plus (Leica Microsystems, Wetzlar, Germany).

### 2.17. Measurement of Acetylcholine (ACh) Level, Acetylcholinesterase (AChE) activity, and Acetyltransferase (ChAT) Activity

After the behavioral studies were finished, all mice were sacrificed and the hippocampus and cortex were carefully dissected from the brains and rapidly stored at -80°C for examination. The hippocampus and cortex tissues were homogenized with ice-cold saline, centrifuged at 12,000 × g for 10 min at 4°C. The supernatants were collected to detect the ACh level, AChE activity, and ChAT activity according to the manufacturer's instructions of the Ach assay kit (cat #A105-1-1), AChE activity assay kit (cat #A024-1-1), and ChAT assay kit (cat #A079-1-1) from Jiancheng Bioengineering Institute (Nanjing, China), respectively.

### 2.18. Total Antioxidant Capacity Assay

The brain tissues of mice in each group were homogenized in cold PBS. A rapid 2′2′-azino-bis-3-ethylbenzthiazoline-6-sulfonic acid (ABTS) method was used to measure the total antioxidant capacity according to the kit manufacturer's instructions from Beyotime Institute of Biotechnology (cat #S0121, Shanghai, China).

### 2.19. Immunohistochemistry

The brain tissues of mice in the control, FA-97- (10 mg/kg), and CAPE- (10 mg/kg) treated groups were immersed in 4% formaldehyde (pH 7.4) for 24 h, embedded in paraffin, and cut into sections 4 mm thick using standard histological techniques to prepare paraffin sections. The expressions of HO-1 and NQO-1 of the brain tissues were assessed using an Immunohistochemistry Application Solutions Kit (ZSGB-BIO, Beijing, China) with specific antibodies (1 : 100).

### 2.20. Statistical Analysis

The data shown in the study were obtained from at least three independent experiments, and all data in different experimental groups were expressed as the mean ± standard deviation (SD). Statistical analyses were performed using a one-way ANOVA, with post hoc analysis. Details of each statistical analysis are provided in the figure legends. Differences with *P* values < 0.05 were considered statistically significant.

## 3. Results

### 3.1. FA-97 Attenuates H_2_O_2_-Induced Cytotoxicity in SH-SY5Y and PC12 Cells

To evaluate the effect of FA-97 on H_2_O_2_-induced cytotoxicity in SH-SY5Y and PC12 cells, cellular morphological observation, CCK8 assay, and LDH release assay were performed. The preliminary experiment revealed that treatment of H_2_O_2_ ranging from 25 *μ*M to 600 *μ*M for 24 h decreased cell viability in a concentration-dependent manner, and H_2_O_2_ at 500 *μ*M induced cell injury in a moderate manner both in SH-SY5Y and PC12 cells ([Supplementary-material supplementary-material-1]). Therefore, this concentration was used for all further experiments. In addition, pretreatment with FA-97 for 24 h alone at 1 *μ*M had no effect on cell viability, and the reduced viability of neuronal cell was observed at 2 *μ*M FA-97 ([Supplementary-material supplementary-material-1]). So the concentration of FA-97 used in our study was no more than 1 *μ*M. As shown in Figure 1(b), cellular morphological observation showed that FA-97 prevented the loss of SH-SY5Y and PC12 cells and reversed the morphological alterations including cell shape, detachment, and shrinkage of cell bodies induced by H_2_O_2_ (500 *μ*M). In addition, the CCK8 assay showed that H_2_O_2_ decreased the viability of SH-SY5Y and PC12 cells, while FA-97 (0.25, 0.5, and 1 *μ*M) enhanced the survival rates of both SH-SY5Y (Figure 1(c)) and PC12 cells (Figure 1(d)). Moreover, FA-97 lowered the LDH release of SH-SY5Y (Figure 1(e)) and PC12 cells (Figure 1(f)) induced by H_2_O_2_ in a concentration-dependent manner. Taken together, FA-97 attenuated H_2_O_2_-induced cytotoxicity in SH-SY5Y and PC12 cells.

### 3.2. FA-97 Inhibits H_2_O_2_-Induced Apoptosis of SH-SY5Y and PC12 Cells

**Figure 2:**
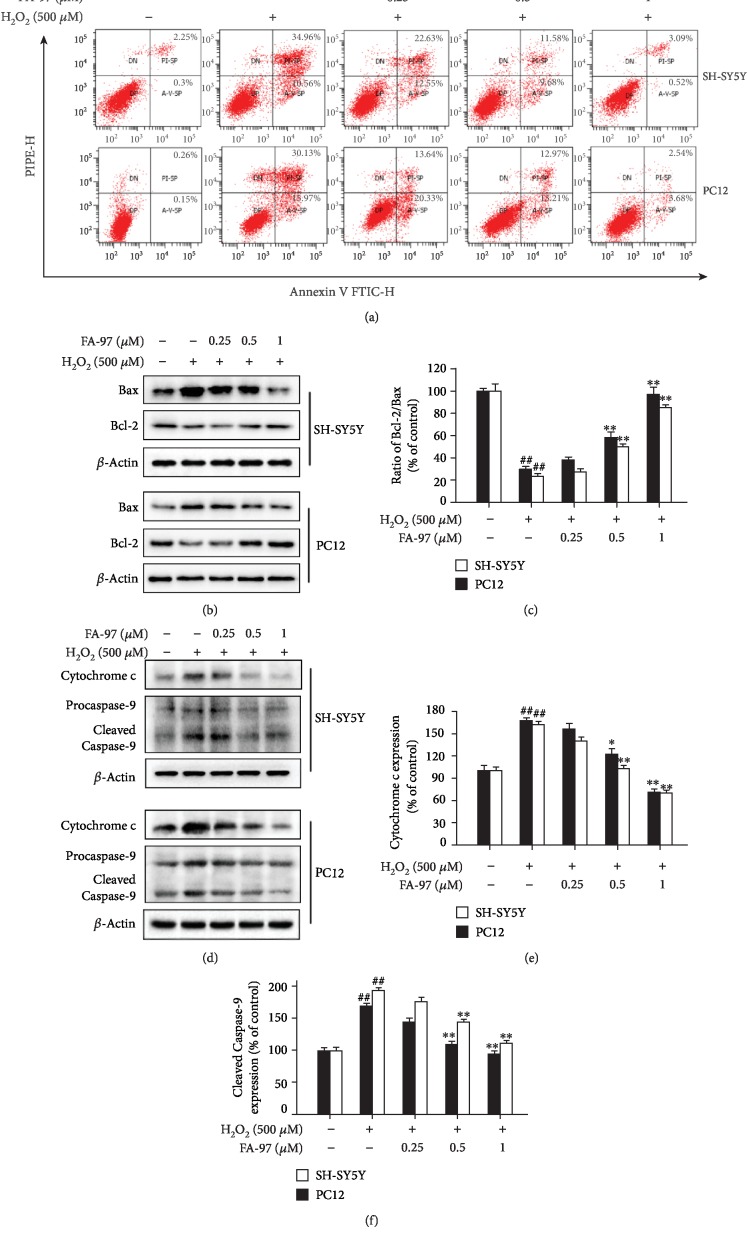
Effect of FA-97 on H_2_O_2_-induced apoptosis of SH-SY5Y and PC12 cells. SH-SY5Y and PC12 cells were treated with H_2_O_2_ (500 *μ*M) and FA-97 (0, 0.25, 0.5, and 1 *μ*M) for 24 h. (a) After being double stained by Annexin V-FITC and PI, the percentage of apoptotic cells was measured by flow cytometry. (b) Protein levels of Bax, Bcl-2, and *β*-actin in total protein lysates were analyzed by Western blot using the indicated antibodies. (c) The Bcl-2/Bax ratios were represented by densitometric analysis, and *β*-actin was used as the loading control. (d) The expressions of Cytochrome c, Caspase-9, and *β*-actin were measured by Western blot analysis. The relative ratios of Cytochrome c (e) and cleaved Caspase-9 (f) were represented by densitometric analysis. Results are representative of three independent experiments and expressed as means ± SD. ^##^*P* < 0.01 compared with the control group; ^∗^*P* < 0.05 and ^∗∗^*P* < 0.01 compared with the H_2_O_2_-stimulated group.

To further study the protective effect of FA-97 on H_2_O_2_-treated SH-SY5Y and PC12 cells, the apoptosis rates and expression of apoptosis-related proteins were detected. Based on the Annexin V/PI staining (Figure 2(a) and [Supplementary-material supplementary-material-1]), the apoptotic cell ratios of SH-SY5Y (45.52 ± 1.84%) and PC12 (46.1 ± 2.27%) were much higher in the presence of H_2_O_2_ compared to the control groups (2.55 ± 0.42% of SH-SY5Y, 0.41 ± 0.02% of PC12, respectively). Treatment of FA-97 decreased the percentage of apoptotic SH-SY5Y and PC12 cells induced by H_2_O_2_ (Figure 2(a) and [Supplementary-material supplementary-material-1]). To confirm the antiapoptotic effect of FA-97 on H_2_O_2_-induced nerve cell apoptosis, the expressions of Bax, Bcl-2, Cytochrome c, and Caspase-9 in SH-SY5Y and PC12 cells were detected by Western blot analysis. As show in Figure 2(b), the proapoptotic protein Bax was upregulated, whereas the antiapoptotic protein Bcl-2 was downregulated with H_2_O_2_ stimulation. However, these effects of H_2_O_2_ were inhibited by FA-97, and the Bcl-2/Bax ratios of both SH-SY5Y and PC12 cells were increased by FA-97 (Figure 2(c)). Correspondingly, FA-97 inhibited the H_2_O_2_-induced expression of Cytochrome c (Figures 2(d) and 2(e)). Compared to the control group, H_2_O_2_ exposure markedly increased the expression of cleaved Caspase-9, while FA-97 reduced the activation of Caspase-9 (Figures 2(d) and 2(f)). These results indicated that FA-97 treatment inhibited H_2_O_2_-induced apoptosis of SH-SY5Y and PC12 cells.

### 3.3. FA-97 Suppresses H_2_O_2_-Induced Oxidative Stress in SH-SY5Y and PC12 Cells

**Figure 3:**
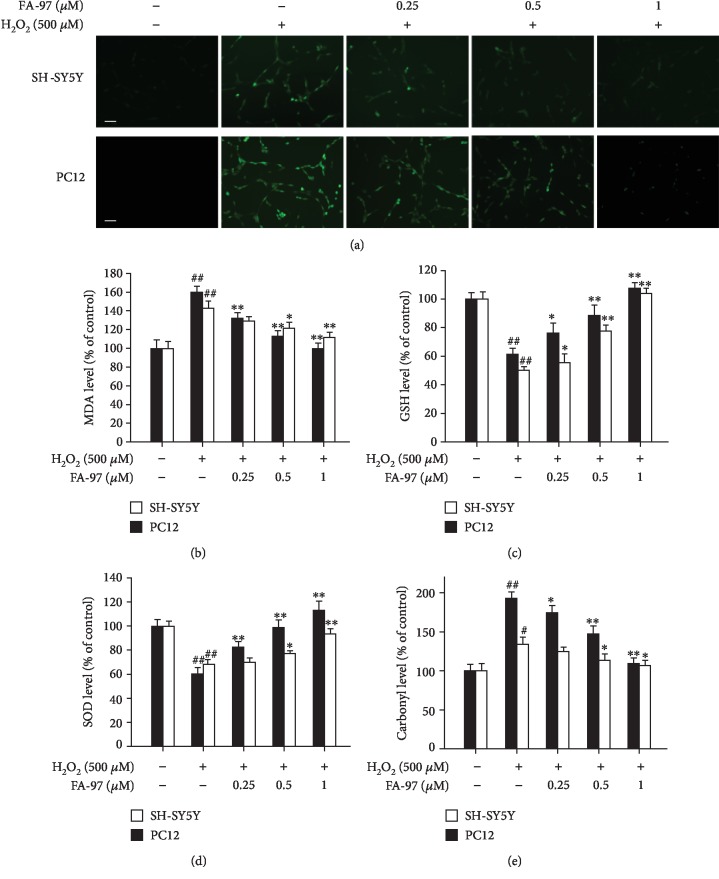
Effect of FA-97 on H_2_O_2_-induced oxidative stress in SH-SY5Y and PC12 cells. SH-SY5Y and PC12 cells were treated with H_2_O_2_ (500 *μ*M) and FA-97 (0, 0.25, 0.5, and 1 *μ*M) for 24 h. (a) Representative images of SH-SY5Y and PC12 cells stained with DCFH-DA (a ROS fluorescence probe). (b–e) The malondialdehyde (MDA) level (b), glutathione (GSH) level (c), superoxide dismutase (SOD) activity (d), and protein carbonylation (e) were measured according to the kit manufacturer's instructions. Scale bars, 200 *μ*m. Results are representative of three independent experiments and expressed as means ± SD. ^#^*P* < 0.05 and ^##^*P* < 0.01 compared with the control group and ^∗^*P* < 0.05 and ^∗∗^*P* < 0.01 compared with the H_2_O_2_-stimulated group.

The overproduction of ROS and superoxide play an important role in H_2_O_2_-inflicted oxidative damage and cytotoxicity [7]; we next evaluated the effect of FA-97 on H_2_O_2_-induced oxidative stress. As shown in Figure 3(a), the DCFH-DA fluorescence induced by H_2_O_2_ in SH-SY5Y and PC12 cells was inhibited by FA-97. Treatment of SH-SY5Y and PC12 cells with H_2_O_2_ alone for 24 h increased intracellular malondialdehyde (MDA) levels, while FA-97 suppressed the MDA level in a concentration-dependent manner (Figure 3(b)). In addition, H_2_O_2_ caused a decrease in the levels of cellular glutathione (GSH) (Figure 3(c)) and superoxide dismutase (SOD) (Figure 3(d)), which were increased by FA-97 both in SH-SY5Y and PC12 cells. Moreover, the protein carbonylation level increased by H_2_O_2_ was inhibited by FA-97 (Figure 3(e)). On the basis of these results, FA-97 suppressed H_2_O_2_-induced oxidative stress in SH-SY5Y and PC12 cells.

### 3.4. FA-97 Activates the Nrf2/HO-1 Pathway in H_2_O_2_-Induced SH-SY5Y and PC12 Cells

**Figure 4:**
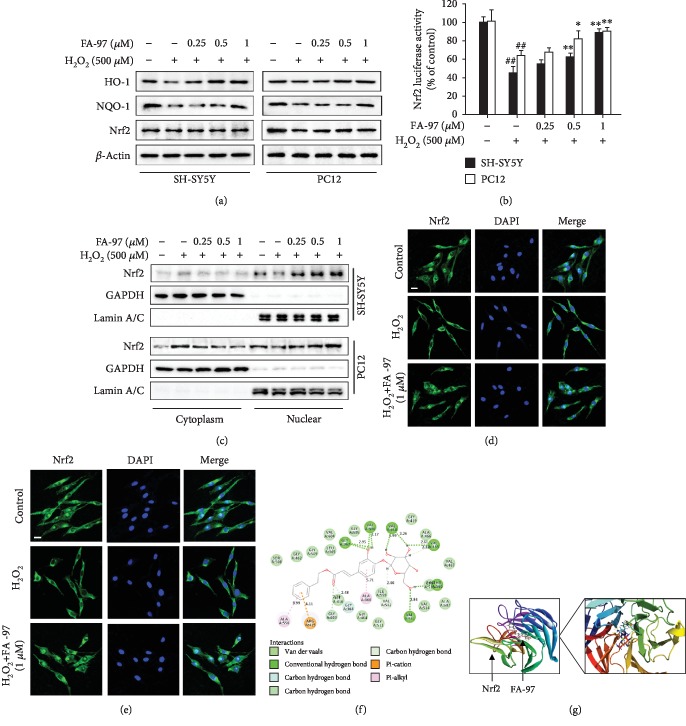
Effect of FA-97 on Nrf2/HO-1 signaling in SH-SY5Y and PC12 cells. SH-SY5Y and PC12 cells were treated with H_2_O_2_ (500 *μ*M) and FA-97 (0, 0.25, 0.5, and 1 *μ*M) for 24 h. (a) The expression of HO-1, NQO-1, Nrf2, and *β*-actin was detected by Western blot. (b) After being transfected with ARE-luciferase reporter plasmid, the Nrf2 transcription activity in SH-SY5Y and PC12 cells was detected by luciferase activity assay. (c) The expressions of Nrf2 in cytosolic and nuclear extracts were determined by Western blot. Lamin A/C and GAPDH were used as nuclear and cytoplasmic markers, respectively. (d, e) SH-SY5Y cell slides (d) and PC12 cell slides (e) were immune-stained with anti-Nrf2 (green) and DAPI (blue), and then the nuclear translocation of Nrf2 was observed by confocal laser-scanning microscope. Scale bars, 15 *μ*m. The results are representative of three independent experiments and expressed as means ± SD. ^##^*P* < 0.01 compared with the control group and ^∗^*P* < 0.05 and ^∗∗^*P* < 0.01 compared with the H_2_O_2_-stimulated group.

It has been reported that Nrf2/HO-1 signaling plays an important role in protecting nerve cells from oxidative damage via inhibiting the intracellular ROS level by inducing phase II detoxifying enzymes including hemeoxygenase-1 (HO-1), quinone oxidoreductase 1 (NQO-1), and glutamate-cysteine ligase (GCL) [15]. We therefore explored the effect of FA-97 on the Nrf2/HO-1 pathway. As expected, Western blot analysis showed that total expressions of HO-1 and NQO-1 in SH-SY5Y and PC12 cells stimulated by H_2_O_2_ were promoted by FA-97, while FA-97 had no effect on the Nrf2 level (Figure 4(a)). Compared with the H_2_O_2_-stimulated group, FA-97 at 1 *μ*M promoted the expression of HO-1 and NQO-1 by 98.4% and 39.9% in SH-SY5Y cells ([Supplementary-material supplementary-material-1]), respectively, and the increased rate of the HO-1 and NQO-1 levels promoted by FA-97 (1 *μ*M) in PC12 cells was 26.4% and 29.2%, respectively ([Supplementary-material supplementary-material-1]). In addition, the transcription activity of Nrf2 was promoted by FA-97 in H_2_O_2_-stimulated SH-SY5Y and PC12 cells which was confirmed by the luciferase reporter assay (Figure 4(b)). Moreover, Western blot for nuclear separation indicated that the nuclear Nrf2 level was increased and the Nrf2 expression in cytoplasm was inhibited by FA-97 treatment (Figure 4(c)). The increased rate of nuclear Nrf2 level in SH-SY5Y and PC12 cells promoted by FA-97 (1 *μ*M) was 114.2% and 70.7%, respectively ([Supplementary-material supplementary-material-1]). Correspondingly, immunofluorescence staining showed that the decreased Nrf2 nuclear translocation was promoted by FA-97 (1 *μ*M) both in SH-SY5Y (Figure 4(d)) and in PC12 cells (Figure 4(e)).

Small-molecule modulators activate the Nrf2 pathway by binding with Nrf2 or Keap1 directly to disrupt the protein-protein interaction between Nrf2 and Keap1 for Nrf2 degradation [35]. To explore how FA-97 promotes the activation and nuclear translocation of Nrf2, we performed a molecular docking simulation to investigate potential interactions of FA-97 and Nrf2. As shown in Figure 4(f), FA-97 was able to combine with Nrf2 under the effect of hydrogen bond and conjugation, and this energy minimized small molecular can stretch into the hydrophobic pocket well. The FA-97 formed a stable hydrogen bond with Gly367 and Val606 on the phenolic hydroxyl group. And the terminal glucose can also form various hydrogen bonds with Nrf2 on Val418, Val465, Val512, Thr560, and Val561 which enhanced the combination effect of the small molecule and the receptor. Taken together, FA-97 is a potential Nrf2 activator in SH-SY5Y and PC12 cells and might bind with Nrf2 directly.

### 3.5. Nrf2 Is Involved in the Antioxidant Effect of FA-97 on Neuronal Cells

**Figure 5:**
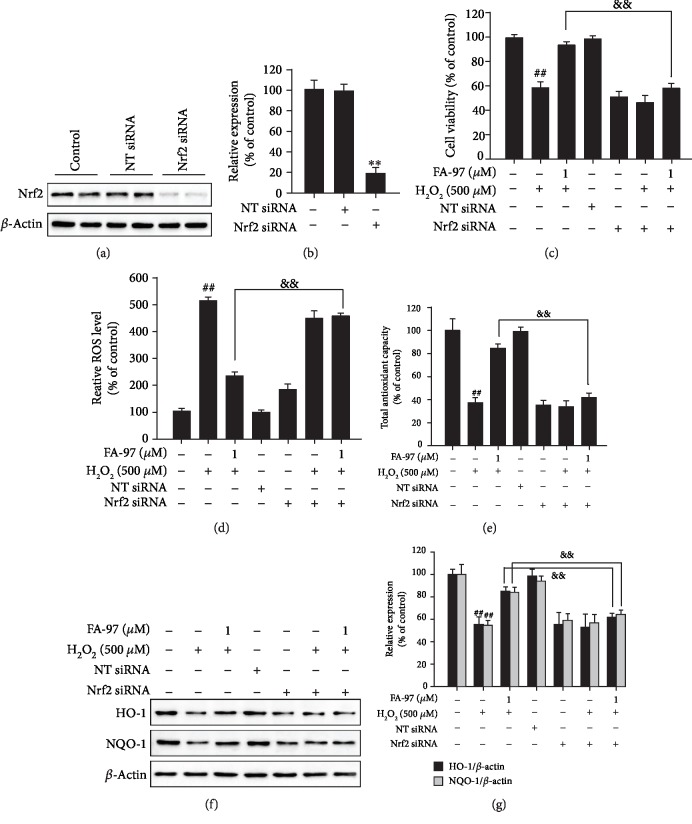
Nrf2 is involved in the antioxidant effect of FA-97 on neuronal cells. Nrf2 siRNA and nontargeting siRNA (NT siRNA) were transfected into SH-SY5Y cells. After being cultured in serum-free medium for 8 h, cells were treated with H_2_O_2_ (500 *μ*M) and FA-97 (0, 0.25, 0.5, and 1 *μ*M) for 24 h. (a) The expression of Nrf2 was detected by Western blot assay. (b) The relative ratio of Nrf2 was represented by densitometric analysis. (c) The viability of SH-SY5Y cells was evaluated by CCK8 assay. (d) SH-SY5Y cells were incubated with DCFH-DA for 30 min at 37°C in the dark, and then the ROS level was measured by spectrofluorometer. (e) The total antioxidant capacity of SH-SY5Y cells was detected according to the kit manufacturer's instruction. (f) The expression of HO-1, NQO-1, and *β*-actin was measured by Western blot analysis. (g) The relative ratios of HO-1 and NQO-1 were represented by densitometric analysis. The results are representative of three independent experiments and expressed as means ± SD. ^#^*P* < 0.05 and ^##^*P* < 0.01 compared with the control group; ^&&^*P* < 0.01 compared with the H_2_O_2_+FA-97-treated group.

To investigate the role of Nrf2 in the antioxidant processes of FA-97, we diminished the expression of Nrf2 in SH-SY5Y cells by Nrf2 siRNA transfection. As expected, the siRNA transfection resulted in the lower expression of Nrf2 in cell lysates (Figures 5(a) and 5(b)). The CCK8 assay showed that the increased survival rates of SH-SY5Y cells by FA-97 were decreased after diminishing the expression of Nrf2 in H_2_O_2_-stimulated SH-SY5Y cells (Figure 5(c)). In addition, the inhibitory effect of FA-97 on H_2_O_2_-induced ROS generation was attenuated by Nrf2 siRNA transfection (Figure 5(d)). Similarly, the total antioxidant capacity of SH-SY5Y cells promoted by FA-97 was inhibited by Nrf2 siRNA (Figure 5(e)). Moreover, Nrf2 siRNA transfection reversed the upregulated HO-1 and NQO-1 of SH-SY5Y cells by FA-97 treatment (Figures 5(f) and 5(g)). These data suggested that FA-97 exerts antioxidant functions by activating Nrf2.

### 3.6. FA-97 Prevents Scopolamine-Induced Learning and Memory Impairments In Vivo

**Figure 6:**
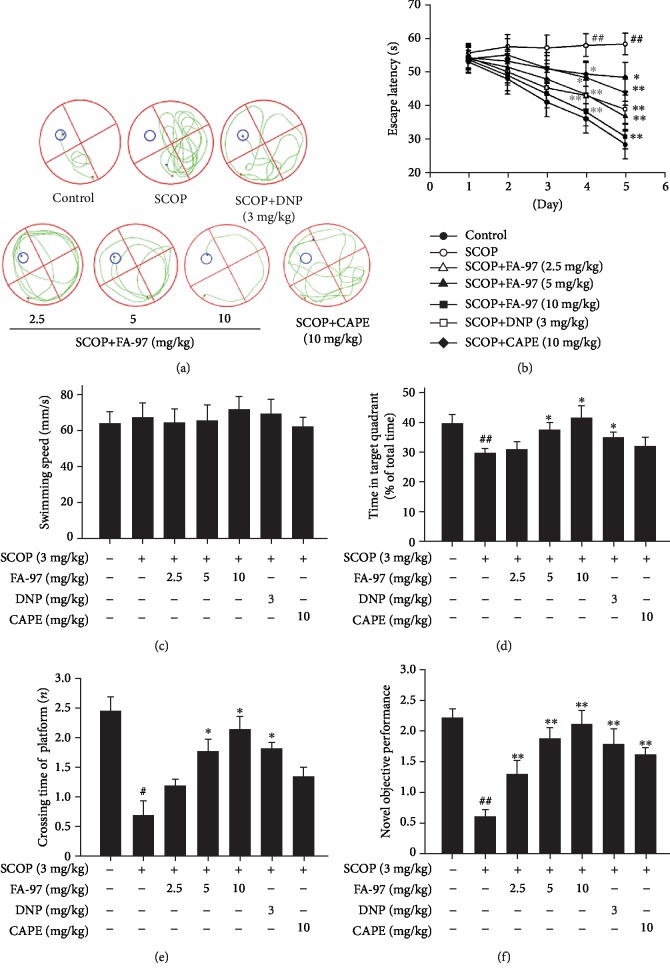
Effect of FA-97 on scopolamine-induced learning and memory impairments. The Morris water maze test was performed. The swimming tracks (a), escape latency of five consecutive days test (b), swimming speed (c), time spent in the target quadrant (d), and crossing times of the platform (e) were shown. (f) The novel object recognition test was performed and the novel objective performance was recorded. Experimental values were expressed as means ± SD. ^#^*P* < 0.05 and ^##^*P* < 0.01 compared with the control group and ^∗^*P* < 0.05 and ^∗∗^*P* < 0.01 compared with the SCOP-treated group.

In order to investigate whether FA-97 could improve the cognitive function *in vivo*, a scopolamine- (SCOP-) induced learning and memory impairment mouse model was used. The Morris water maze test was performed to evaluate the effect of FA-97 on spatial memory. As shown in Figure 6(a), compared with the control group, the swimming track of mice in the SCOP group on the fifth experimental day is complex and mice swim aimlessly to find the hidden platform, which suggested that intraperitoneal injection with SCOP (3 mg/kg) induces the impairment of spatial memory. Compared with the SCOP group, FA-97 treatment improved the spatial memory of mice in a dose-dependent manner and mice in FA-97 (10 mg/kg) swim to the platform directly. In addition, the escape latency (swimming time for mice to find the platform) is reduced progressively during the five training days (Figure 6(b)). The escape latency is longer than the control group from the second to the fifth day, while mice in the FA-97-treated groups exhibited an improved performance. Compared with the control group, SCOP (3 mg/kg), FA-97 (2.5, 5, 10 mg/kg), CAPE (10 mg/kg), or DNP (3 mg/kg) treatment had no effect on the average swimming speed (Figure 6(c)). In the spatial probe trial, time spend in the target quadrant of mice in the FA-97- (5, 10 mg/kg) or DNP- (3 mg/kg) treated group was longer than the SCOP-treated group (Figure 6(d)). In addition, compared with the control group, the time spent in crossing the platform of the SCOP group was shorter (Figure 6(e)). However, compared to the SCOP-treated group, FA-97 (5, 10 mg/kg) or DNP (3 mg/kg) treatment increased the crossing time significantly. In the novel object recognition test, SCOP-treated mice showed a lower level of discrimination index, while FA-97, DNP, and CAPE improved the novel objective performance of mice (Figure 6(f)). These results indicated that treatment with FA-97 is beneficial for SCOP-induced cognitive impairment.

### 3.7. FA-97 Reduces Neuronal Apoptosis and Protects against Cholinergic System Dysfunction in Scopolamine-Treated Mouse Brain

**Figure 7:**
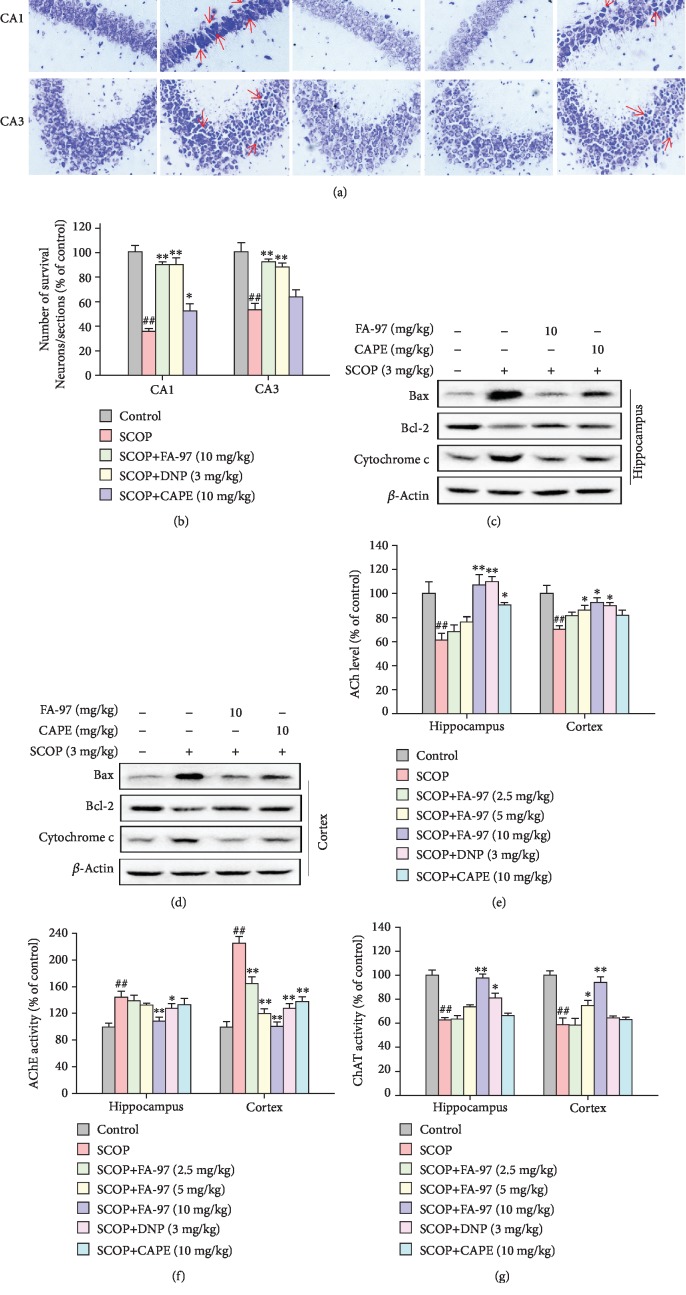
Effect of FA-97 on the neuron function and cholinergic system in scopolamine-treated mouse brain. (a) Representative images of the Nissl-stained neurons in CA1 and CA3 areas are shown. (b) The quantitative analysis of the relative number of survival neurons in each section based on the Nissl-staining assay. (c, d) The expressions of Bax, Bcl-2, and Cytochrome c in the hippocampus and cortex were measured by Western blot analysis. (e–g) The relative acetylcholine (ACh) level (e), acetylcholinesterase (AChE) activity (f), and acetyltransferase (ChAT) activity (g) in the hippocampus and cortex were measured according to the kit manufacturer's instructions. The results are representative of three independent experiments and expressed as means ± SD. ^#^*P* < 0.05 and ^##^*P* < 0.01 compared with the control group; ^∗^*P* < 0.05 and ^∗∗^*P* < 0.01 compared with the SCOP-treated group.

To illuminate the protective effect of FA-97, we detected the degree of neuronal apoptosis and indexes of cholinergic system in SCOP-treated mice. Nissl staining showed that SCOP administration alone reduced the density of healthy neuron cells in the CA1 and CA3 areas of the hippocampus and decreased the amount of surviving neuronal cells (Figures 7(a) and 7(b)), as well as resulted in typical neuropathological changes, including Nissl body loss and nucleus shrinkage or disappearance. However, FA-97 promoted neuron survival and prevented SCOP-induced neuronal loss in the CA1 and CA3 areas. In addition, the expression of various apoptotic and antiapoptotic markers in the SCOP-treated mouse brain was examined by Western blot. As shown in Figures 7(c) and 7(d), levels of Bax and Cytochrome c in the SCOP-treated group were significantly higher than those in the control group and SCOP downregulated the expression of Bcl-2. FA-97 reduced the amount of Bax and Cytochrome c, as well as increased Bcl-2 both in the hippocampus and cortex. Compared with SCOP-treated group, FA-97 treatment resulted in a 2.4-fold increase in the ratio of Bcl-2/Bax in the hippocampus and a 1.5-fold increase in the cortex ([Supplementary-material supplementary-material-1]), and the inhibition rate of Cytochrome c by FA-97 was 44.2% and 41.5% in the hippocampus and cortex, respectively ([Supplementary-material supplementary-material-1]). Furthermore, FA-97 increased the acetylcholine (ACh) contents decreased by SCOP administration in the hippocampus and cortex (Figure 7(e)). As shown in Figure 7(f), the activity of acetylcholinesterase (AChE) in the SCOP-treated group was increased, whereas FA-97 and DNP decreased the activities of AChE significantly. The choline acetyltransferase (ChAT) activities inhibited by SCOP in both the hippocampus and cortex were promoted by FA-97 remarkably (Figure 7(g)). These results indicated that FA-97 reduced neuronal apoptosis and protected against cholinergic system dysfunction induced by SCOP.

### 3.8. FA-97 Protects against Oxidative Stress and Activates Nrf2 in Scopolamine-Treated Mice

**Figure 8:**
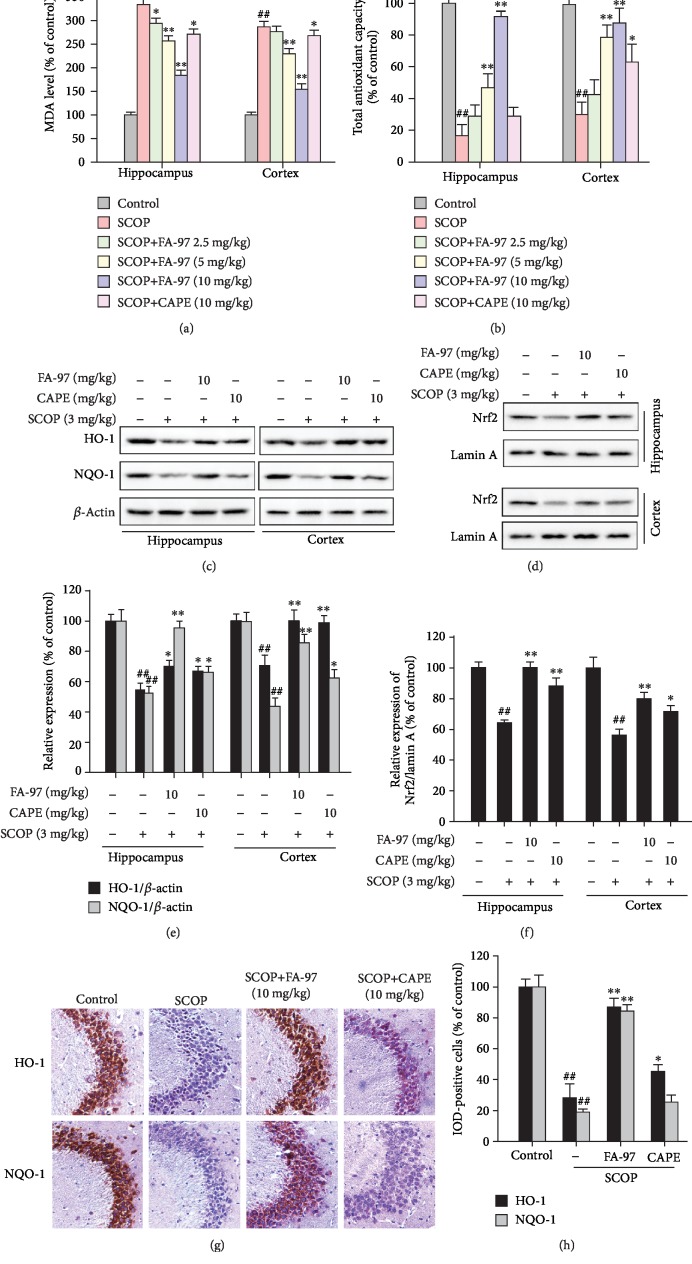
Effect of FA-97 on scopolamine-induced oxidative stress and the Nrf2 pathway in vivo. Brain tissues were homogenized in cold PBS. (a, b) The levels of MDA (a) and total antioxidant capacity (b) in the hippocampus and cortex were measured according to the kit manufacturer's instructions. (c) The expressions of HO-1, NQO-1, and *β*-actin in the hippocampus and cortex were measured by Western blot analysis. (d) The nuclear Nrf2 expressions in the hippocampus and cortex were detected. The relative expressions of HO-1, NQO-1 (e), and Nrf2/Lamin A (f) were represented by densitometric analysis. (g) The expressions of HO-1 and NQO-1 in brain sections were detected by immunohistochemistry (IHC), and the positive cells were brown. (h) Quantification of IHC images was by Image-Pro Plus software, and 10 fields were counted for each mouse. IOD of HO-1- and NQO-1-positive cells were shown. The results are representative of three independent experiments and expressed as means ± SD. ^##^*P* < 0.01 compared with the control group; ^∗^*P* < 0.05 and ^∗∗^*P* < 0.01 compared with the SCOP-treated group.

To further investigate the potential mechanisms of FA-97 *in vivo*, oxidative stress in SCOP-treated mice was evaluated. Therefore, the MDA level and total antioxidant capacity of the hippocampus and cortex in SCOP-treated mouse brains were detected initially. As shown in Figure 8(a), SCOP increased the MDA levels in the hippocampus and cortex, while these effects were reversed by FA-97. Moreover, FA-97 increased the total antioxidant capacity in both the hippocampus and cortex of SCOP-treated mouse brains (Figure 8(b)). Based on the activation effect of FA-97 on Nrf2 *in vitro*, we next explored whether FA-97 can activate the Nrf2 pathway *in vivo*. Western blot analysis (Figure 8(c)) showed that total expressions of HO-1 and NQO-1 in the hippocampus and cortex were upregulated by FA-97 (10 mg/kg), which was also supported by the results of immunohistochemistry (Figures 8(g) and 8(h)). Compared with the SCOP-treated group, FA-97 treatment resulting in HO-1 expression in the hippocampus and cortex increased by 27.6% and 40.4%, and the increase rate of NQO-1 in the hippocampus and cortex was 83.3% and 90.7%, respectively (Figure 8(e)). Moreover, Western blot for nuclear separation indicated that the nuclear Nrf2 level was increased in the hippocampus and cortex of SCOP-treated mouse brains (Figure 8(d)). Compared with the SCOP-treated group, the increase rate of Nrf2/Lamin A in the hippocampus and cortex by FA-97 was 57.0% and 43.1%, respectively (Figure 8(f)). Taken together, FA-97 protects against oxidative stress and activates Nrf2 in SCOP-treated mice.

## 4. Discussion

Up to now, only four cholinesterase inhibitors and memantine have shown sufficient safety and efficacy and have been approved for clinical use in AD [36]. The amyloid beta (A*β*) and hyperphosphorylated tau protein are two key constituents of plaques and neurofibrillary tangles (NFTs) involved in the pathogenesis of AD [37]. Over the last decade, more than 50 drug candidates targeting A*β* or tau protein have successfully passed phase II clinical trials, but none has passed a phase III clinical trial, as the precise molecular mechanisms of AD are still not fully understood [36]. Hence, effective agents acting on other molecular targets involved in the pathogenesis of AD and innovative treatment strategies for AD are urgently needed.

In this study, a new synthetic caffeic acid phenethyl ester (CAPE) derivative (caffeic acid phenethyl ester 4-*O*-glucoside, FA-97) is synthesized (Figure 1(a)) and proved to protect against oxidative stress-mediated neuronal cell apoptosis *in vitro* and scopolamine-induced cognitive impairment *in vivo*. CAPE, a natural phenolic compound derived from honeybee hive propolis, has been widely reported to possess neuroprotective effects and improve learning and memory ability in AD mice, which could be a potential therapeutic agent against AD [27–29, 38, 39]. However, the unstable chemical property, low water solubility, and poor bioavailability of CAPE limit its efficacy, and its half-life is 20-28 minutes and independent of the dose after intragastric administration [34]. Therefore, FA-97 is newly synthesized by introducing a D-glucose into CAPE to construct a glucosidic bond and to enhance the water solubility of this compound. Caffeic acid 4-*O*-glucoside ([Supplementary-material supplementary-material-1]), which is extracted of *Drynaria fortunei* rhizomes, a widely distributed traditional medicine, has been reported to recover A*β*_25-35_-induced axonal atrophy in cultured cortical neurons [40]. In the light of the pharmacophore combination principle in medicinal chemistry, the two different functional groups of CAPE and caffeic acid 4-*O*-glucoside can be connected with a linker to form a new compound, FA-97, which is supposed to increase the bioactivities and water solubility. According to the neuroprotective activities of both CAPE and caffeic acid 4-*O*-glucoside, the effect of FA-97 on H_2_O_2_-induced apoptosis of SH-SY5Y and PC12 cells was investigated initially. As a result, we found that FA-97 inhibited H_2_O_2_-induced cytotoxicity and apoptosis both in SH-SY5Y and PC12 cells by CCK8 cell viability test, LDH level detection, Annexin V/PI staining, and Western blot assay. All of these results indicate that FA-97 has the neuroprotective effect *in vitro*.

It is strongly evident that oxidative stress has been recognized as a contributor in the pathogenesis of AD [3, 12]. Neuronal cells are more vulnerable to free radical damage due to high oxygen consumption and lack of antioxidant enzyme availability compared to other organs [41]. Signs of increased oxidative stress are apparent in tissue samples taken from AD patients, with evidence in the diseases for protein modifications induced directly by ROS or indirectly by lipid peroxidation products [10, 11]; a high level of a serum peroxidation marker was found in 101 patients associated with an increased risk of AD [42]. Recent experiments suggested that during the early stage of the AD, A*β* could enter the mitochondria to increase the generation of free radicals and induce oxidative stress [43]; the ROS burst was mainly the result of impaired axonal transport and energy dysfunction of mitochondria caused by an abnormally phosphorylated tau protein [44]; the high concentration of redox-active copper and iron is consistent with their catalytic action in Fenton chemistry to form reactive hydroxyl radicals which may cause damage to biomolecules in the brain, including DNA [8]. Meanwhile, many compounds accepted for the treatment of AD were found to possess potent antioxidant properties such as selegiline, piracetam, flavonoids, melatonin, and carotenoid [3]. Therefore, the effect of FA-97 on H_2_O_2_-induced oxidative stress in SH-SY5Y and PC12 cells was detected. We found that FA-97 suppressed the ROS level in H_2_O_2_-induced neuronal cells detected by the DCFH-DA fluorescence probe. Moreover, the activity of several main antioxidant enzymes (SOD and GSH) were increased by FA-97 markedly, while the level of prooxidants (MDA and carbonyl) both in H_2_O_2_-induced SH-SY5Y and in PC12 cells was inhibited by FA-97. On the basis of these results, FA-97 can suppress H_2_O_2_-induced oxidative stress in neuronal cells and antioxidation action may be involved in the neuroprotective effect of FA-97.

The Nrf2/HO-1 pathway is a critical pathway in maintaining cellular redox homeostasis [15]. A connection between Nrf2 deficiency and neurodegeneration, as well as an emerging target against oxidative stress in AD being given by the Nrf2/HO-1 pathway, is supported by a growing body of evidence [15–23]. To elucidate the molecular mechanism of the neuroprotective effect of FA-97, the influence of FA-97 on the Nrf2/HO-1 pathway was explored. We found that FA-97 promoted the transcription activity of Nrf2 in a concentration-dependent manner. As expected, HO-1 and NQO-1, two important downstream proteins of Nrf2, were upregulated by FA-97. Interestingly, FA-97 promoted the expression of Nrf2 in nuclear, while it almost had no effect on the total protein level of Nrf2. These results indicated that the facilitated nuclear translocation which is a key step in the course of FA-97 activates the transcription activity of Nrf2. To translocate into the nuclear, Nrf2 have to dissociate from the Nrf2-Keap1 heterodimer [14]; therefore, Nrf2 activators work effectively by competing with Keap1 to bind with Nrf2 directly [35]. Then, the molecular docking simulation was performed to investigate potential interactions of FA-97 and Nrf2. We found that FA-97 formed a stable hydrogen bond with Gly367 and Val606 on the phenolic hydroxyl group. Meanwhile, the terminal glucose of FA-97 can also form various hydrogen bonds with Nrf2 to enhance the combination effect. Taken together, FA-97 could activate Nrf2 by binding with it directly.

To further investigate the role of Nrf2 in the neuroprotective and antioxidant effects of FA-97, Nrf2 siRNA was then used in our study. As a result, the increased survival rates, inhibited ROS generation, and the promoted total antioxidant capacity, as well as the upregulated HO-1 and NQO-1 in SH-SY5Y cells treated by FA-97, were all reversed after transfection with Nrf2 siRNA. Taken together, these results indicate that FA-97 may be a potential Nrf2 activator by binding with it directly to protect neuronal cells against oxidative stress-mediated cytotoxicity and apoptosis.

The deficiency in the neurotransmitter acetylcholine (ACh) caused by cholinergic malfunction is a key event in AD pathogenesis, which appears in the aged and demented central nervous system [45]. In young and healthy subjects, the cognitive impairment can be artificially induced by blocking cholinergic mechanism [46]. Scopolamine (SCOP), a nonselective muscarinic acetylcholine receptor antagonist, has been reported to induce learning and memory impairments by inhibiting the cholinergic system in the central nervous system, which is regarded as “scopolamine dementia” [47]. Thus, we employed a mimic AD model by treating mice with SCOP to evaluate the anti-AD effect of FA-97. In agreement with other reports [48, 49], the cognitive dysfunction in the short-/long-term, spatial learning, and memory ability were observed in SCOP-treated mice by the Morris water maze (MWM) task and the new object location recognition (OLR) test, while FA-97 can protect against SCOP-induced cognitive impairment. In the cholinergic system, the choline acetyltransferase (ChAT) is the most important synthetic enzyme triggering the synthesis of Ach and acetylcholinesterase (AChE) is a hydrolytic enzyme that hydrolyzes ACh rapidly [50]. In the current study, we found that FA-97 promoted the ACh content and ChAT activity, while inhibiting the activity of AChE both in the hippocampus and cortex areas of SCOP-treated mice. Donepezil (DNP), used as positive control to contrast scopolamine damage, shows similar effect with FA-97. However, CAPE modulated the ACh content and AChE activity weakly and even had no effect on the activity of ChAT.

It has reported that SCOP could induce oxidative stress resulting in neuron injury and apoptosis in the brain of mice [51, 52]. We found that FA-97 markedly attenuates SCOP-induced neuronal apoptosis with the downregulation of the apoptotic index Bax/Bcl-2 and Cytochrome c expressions in the hippocampus and cortex of SCOP-treated mice. In addition, the levels of MDA were inhibited by FA-97 significantly and the total antioxidant capacities in SCOP-treated mice were increased by FA-97 obviously. Therefore, FA-97 could provide a neuroprotective effect against SCOP-induced cholinergic system dysfunction. Moreover, to explore the molecular mechanisms of FA-97 *in vivo*, effects of FA-97 on the Nrf2/HO-1 signaling pathway were tested. As expected, the expressions of HO-1 and NQO-1, as well as the Nrf2 level in the nuclear, were all upregulated by FA-97.

**Figure 9:**
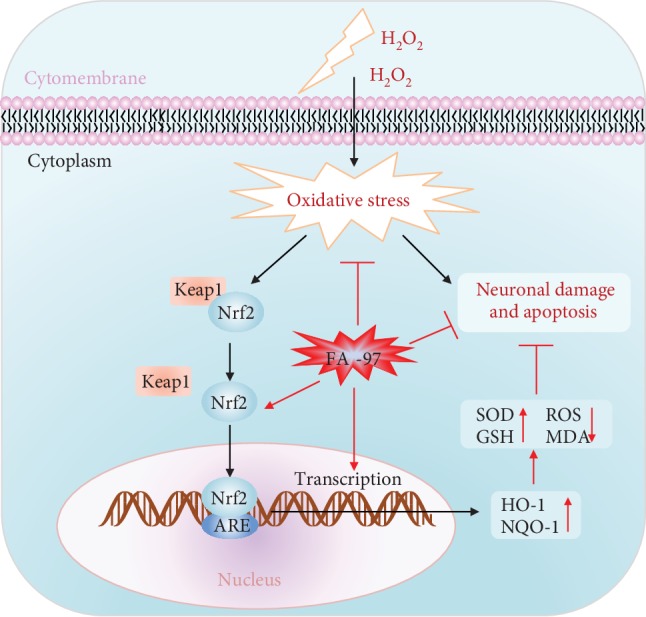
Proposed mechanistic model of FA-97 protects against oxidative stress-mediated neuronal damage and apoptosis. Our findings demonstrated a scenario where FA-97 promotes the nuclear translocation of Nrf2 and the expression of its downstream target proteins HO-1 and NQO-1, to increase the SOD and GSH level, reduce the ROS and MDA level, and enhance the oxidant resistance, and eventually protects against oxidative stress-mediated neuronal cell apoptosis and scopolamine-induced cognitive impairment.

In the present study, we elucidate that FA-97, a new synthetic CAPE derivative, protects against oxidative stress-mediated apoptosis in SH-SY5Y and PC12 cells and scopolamine-induced cognitive impairment by activating Nrf2/HO-1 signaling. Our findings demonstrated a scenario where FA-97 promotes the nuclear translocation of Nrf2 and the expression of its downstream target proteins HO-1 and NQO-1, to reduce the ROS level, enhance the oxidant resistance, and eventually protect against oxidative stress-mediated neuronal cell apoptosis and scopolamine-induced cognitive impairment (Figure 9). However, the present study has some limitations. Except for the SCOP-treated mimic AD model, the effects of FA-97 on transgenic AD mouse models should be evaluated in our further exploration, as well as whether FA-97 could intervene in other signaling pathways or whether Nrf2 is the direct target of FA-97 requires further study.

In conclusion, we successfully confirmed the neuroprotective properties of FA-97, a new synthetic CAPE derivative, protecting against oxidative stress-mediated neuronal cell apoptosis *in vitro* and scopolamine-induced cognitive impairment *in vivo*. This effect is associated with the inhibition of oxidative stress via the activation of Nrf2/HO-1 signaling. FA-97 could be a potential therapeutic agent as a neuroprotective agent against progressive AD.

## Data Availability

All data used to support the findings of this study are included within the article and the supplementary information file.

## Abbreviations

CAPE: Caffeic acid phenethyl ester
AD: Alzheimer's disease
ROS: Reactive oxygen species
H_2_O_2_: Hydrogen peroxide
DNP: Donepezil
SCOP: Scopolamine
LDH: Lactate dehydrogenase
SOD: Superoxide dismutase
MDA: Malondialdehyde
GSH: Glutathione
Nrf2: Nuclear factor erythroid 2-related factor 2
Keap1: Kelch-like ECH-associated protein 1
HO-1: Hemeoxygenase-1
NQO-1: Quinone oxidoreductase-1
AChE: Acetylcholinesterase
ChAT: Acetyltransferase
ACh: Acetylcholine.

## Supplementary Materials

Supplementary Materials Figure S1 shows the timeline of the animal experimental procedure. The scheme is used to illustrate the animal experimental proposal for studying the effect of FA-97 on scopolamine-induced learning and memory impairments in vivo. This figure is related to Section 2.3. Figure S2 shows the viability of SH-SY5Y and PC12 cells treated with H_2_O_2_ at concentrations ranging from 25 *μ*M to 600 *μ*M for 24 h. This result was used to explain why H_2_O_2_ at 500 *μ*M was chosen in our study, which was related to Figure 1 and described in Section 3.1. Figure S3 shows the viability of SH-SY5Y and PC12 cells treated with FA-97 (0, 0.125, 0.25, 0.5, 1, 2, and 3 *μ*M) for 24 h. The reduced viability of neuronal cell was observed at FA-97 (2 *μ*M). This result was used to explain why the concentration of FA-97 used in our study was no more than 1 *μ*M, which was related to Figure 1 and described in Section 3.1. Figure S4 shows the apoptosis rate of SH-SY5Y and PC12 cells treated with H_2_O_2_ and FA-97 for 24 h. This result indicated that FA-97 decreased H_2_O_2_-induced apoptosis of SH-SY5Y and PC12 cells. The histogram is related to Figure 2 and mentioned in Section 3.2. Figure S5 shows the expression of HO-1, NQO-1, and Nrf2 in SH-SY5Y cells. This result indicated that the total expression of HO-1 and NQO-1 was promoted by FA-97, while FA-97 had no effect on the Nrf2 expression. The histogram is related to Figure 4 and is described in Section 3.4. Figure S6 shows the expression of HO-1, NQO-1, and Nrf2 in PC12 cells. This result indicated the total expression of HO-1 and NQO-1 was promoted by FA-97, while FA-97 had no effect on the Nrf2 expression. The histogram is related to Figure 4 and described in Section 3.4. Figure S7 shows Nrf2 expression in the cytoplasm and nuclear of SH-SY5Y and PC12 cells. This result indicated that the nuclear Nrf2 level was increased, while the Nrf2 expression in the cytoplasm was inhibited by FA-97. The histogram is related to Figure 4 and described in Section 3.4. Figure S8 shows the Bcl-2/Bax ratio in the hippocampus and cortex of mice in each group. This result indicated FA-97 increased the Bcl-2/Bax ratio in the SCOP-treated mouse brain. The histogram is related to Figure 7 and described in Section 3.7. Figure S9 shows the Cytochrome c expression in hippocampus and cortex of mice in each group. This result indicated FA-97 reduced the amount of Cytochrome c the in SCOP-treated mouse brain. The histogram is related to Figure 7 and described in Section 3.7. Figure S10 shows the chemical structures of caffeic acid 4-O-glucoside, caffeic acid phenethyl ester (CAPE), and caffeic acid phenethyl ester 4-O-glucoside (FA-97). This is used to compare differences among the three compounds and to help describe the synthetic method of FA-97, by which the two different functional groups of CAPE and caffeic acid 4-O-glucoside can be connected with a linker to form a new compound, in the light of pharmacophore combination principle in medicinal chemistry. All of these have been discussed in Discussion.
