# Supplementary material for: FA-97, a New Synthetic Caffeic Acid Phenethyl Ester Derivative, Protects against Oxidative Stress-Mediated Neuronal Cell Apoptosis and Scopolamine-Induced Cognitive Impairment by Activating Nrf2/HO-1 Signaling
Source: Oxid Med Cell Longev. 2019 Dec 3;2019:8239642. doi: 10.1155/2019/8239642 (PMC6914945; doi:10.1155/2019/8239642)
Supplement: Supplementary Materials — Figure S1 shows the timeline of the animal experimental procedure. The scheme is used to illustrate the animal experimental proposal for studying the effect of FA-97 on scopolamine-induced learning and memory impairments in vivo. This figure is related to Section 2.3. Figure S2 shows the viability of SH-SY5Y and PC12 cells treated with H2O2 at concentrations ranging from 25 μM to 600 μM for 24 h. This result was used to explain why H2O2 at 500 μM was chosen in our study, which was related to Figure 1 and described in Section 3.1. Figure S3 shows the viability of SH-SY5Y and PC12 cells treated with FA-97 (0, 0.125, 0.25, 0.5, 1, 2, and 3 μM) for 24 h. The reduced viability of neuronal cell was observed at FA-97 (2 μM). This result was used to explain why the concentration of FA-97 used in our study was no more than 1 μM, which was related to Figure 1 and described in Section 3.1. Figure S4 shows the apoptosis rate of SH-SY5Y and PC12 cells treated with H2O2 and FA-97 for 24 h. This result indicated that FA-97 decreased H2O2-induced apoptosis of SH-SY5Y and PC12 cells. The histogram is related to Figure 2 and mentioned in Section 3.2. Figure S5 shows the expression of HO-1, NQO-1, and Nrf2 in SH-SY5Y cells. This result indicated that the total expression of HO-1 and NQO-1 was promoted by FA-97, while FA-97 had no effect on the Nrf2 expression. The histogram is related to Figure 4 and is described in Section 3.4. Figure S6 shows the expression of HO-1, NQO-1, and Nrf2 in PC12 cells. This result indicated the total expression of HO-1 and NQO-1 was promoted by FA-97, while FA-97 had no effect on the Nrf2 expression. The histogram is related to Figure 4 and described in Section 3.4. Figure S7 shows Nrf2 expression in the cytoplasm and nuclear of SH-SY5Y and PC12 cells. This result indicated that the nuclear Nrf2 level was increased, while the Nrf2 expression in the cytoplasm was inhibited by FA-97. The histogram is related to Figure 4 and described in Section 3.4. Figure [file 8239642.f1.pdf]

Supplementary Data

Title: FA-97, a new synthetic caffeic acid phenethyl ester derivative, protects against oxidative stress-mediated neuronal cell apoptosis and scopolamine-induced cognitive impairment by activating Nrf2/HO-1 signaling

***To whom correspondence should be addressed:**

Yujie Huang, PhD

Institute of Clinical Pharmacology

Guangzhou University of Chinese Medicine.

No 12, Jichang Road, Guangzhou, Guangdong 510006, China.

Email: huangyujie@gzucm.edu.cn

Phone: 86-20-36585404

Supplementary data contains three Supplementary figures (Figure S1-S10).

Figure S1.

Figure S1 shows the timeline of the animal experimental procedure. The scheme is used to illustrate the animal experimental proposal for studying the effect of FA-97 on scopolamine-induced learning and memory impairments *in vivo*. This figure is related to the methods section (2.3) on page 6 in the manuscript.

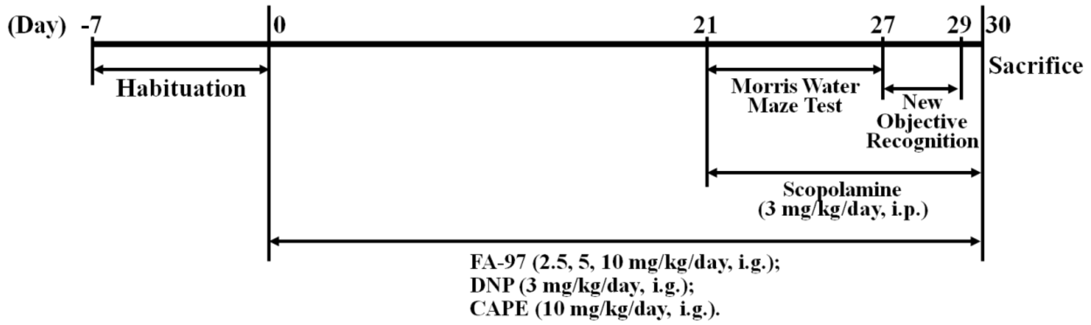

Figure S1. The scheme of animal experimental design. Donepezil (DNP); caffeic acid phenethyl ester (CAPE); intragastric administration (i.g.); intraperitoneal injection (i.p.).

Figure S2.

Figure S2 shows the viability of SH-SY5Y and PC12 cells treated with H_2O_2 at concentrations ranging from 25 μM to 600 μM for 24 h. This result was used to explain why H_2O_2 at 500 μM was chosen in our study, which was related to Figure 1 and described in the results section (3.1) on page 12 line 6-9 of the manuscript.

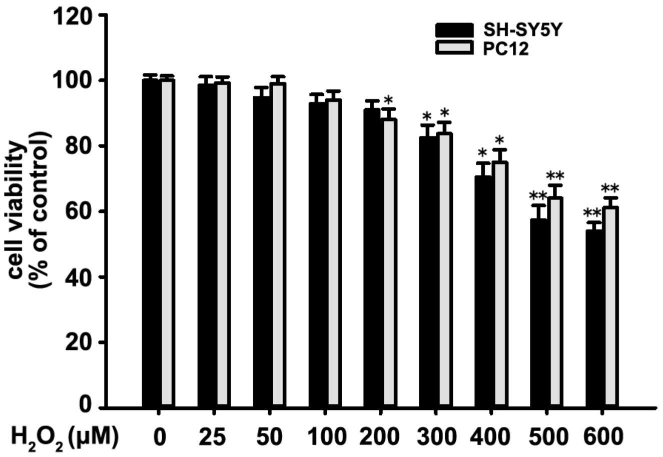

Figure S2. The effect of H_2O_2 at different concentrations on the viability of SH-SY5Y and PC12 cells. Cells were plated into 96-well plates (2×10^5 cells/well) with fresh medium overnight, then treated with H_2O_2 (0, 25, 50, 100, 200, 300, 400, 500 and 600 μM) for 24 h. The cell viability was detected by CCK8 assay. The experiment was performed three times and data are presented as mean ± SD. **P* < 0.05, ***P* < 0.01 compared with control group.

Figure S3.

Figure S3 shows the viability of SH-SY5Y and PC12 cells treated with FA-97 (0, 0.125, 0.25, 0.5, 1, 2 and 3 μM) for 24 h. The reduced viability of neuronal cell was observed at FA-97 (2 μM). This results was used to explain why the concentration of FA-97 used in our study was no more than 1 μM, which was related to Figure 1 and described in the results section (3.1) on page 12 line 9-12 of the manuscript.

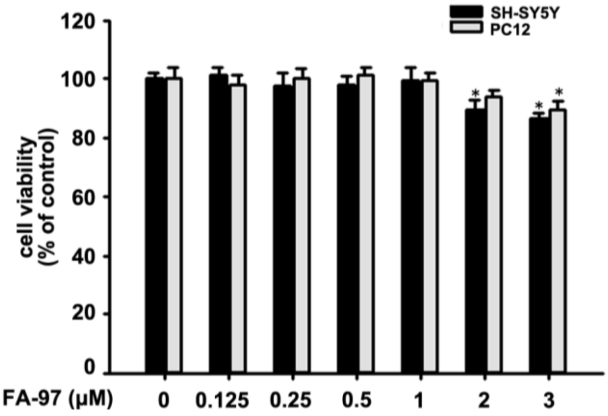

Figure S3. Effects of FA-97 on cell viability of SH-SY5Y and PC12. Cells were plated into 96-well plates (2×10^5 cells/well) with fresh medium overnight, then treated with FA-97 (0, 0.125, 0.25, 0.5, 1, 2, 3 μM) for 24 h. Cell viability was detected by CCK8 assay. Experiment was performed at least three times and data are presented as mean ± SD. $^*P < 0.05$ compared with control group.

Figure S4.

Figure S4 shows the apoptosis rate of SH-SY5Y and PC12 cells treated with H_2O_2 and FA-97 for 24 h. This result indicated FA-97 decreased H_2O_2-induced apoptosis of SH-SY5Y and PC12 cells. The histogram is related to Figure 2 and mentioned in the results section (3.2) on page 12 line 24 of the manuscript.

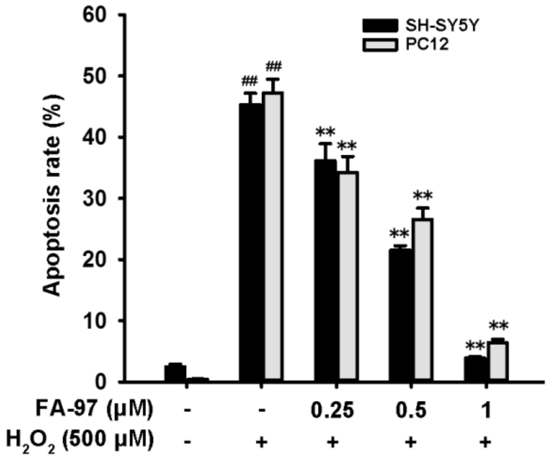

Figure S4.The effect of FA-97 on H_2O_2-induced SH-SY5Y and PC12 cells apoptosis. Cells were treated with H_2O_2 (500 μM) and FA-97 (0, 0.25, 0.5, 1 μM) for 24 h. After double stained by Annexin V-FITC and PI, the percentage of apoptotic cells was measured by flow cytometry. The experiment was performed at least three times and data are presented as mean ± SD. $^{##}P < 0.01$ compared with control group, $^{**}P < 0.01$ compared with H_2O_2-stimulated group.

Figure S5.

Figure S5 shows the expression of HO-1, NQO-1 and Nrf2 in SH-SY5Y cells. This result indicated the total expression of HO-1 and NQO-1 was promoted by FA-97, while FA-97 had no effect on Nrf2 expression. The histogram is related to Figure 4 and described in the results section (3.4) on page 13 line 28-30 of the manuscript.

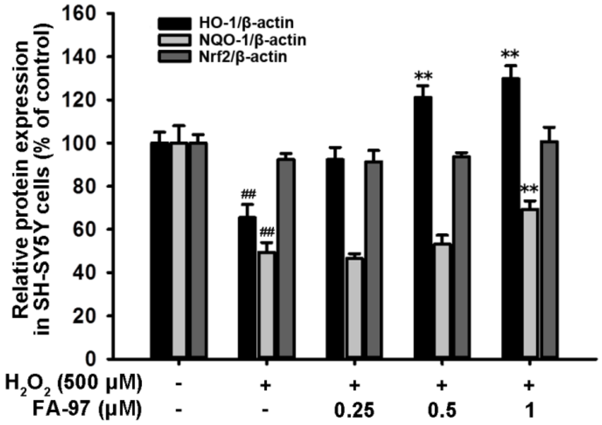

Figure S5. The effect of FA-97 on the expression of HO-1, NQO-1 and Nrf2 in SH-SY5Y cells. Cells were treated with H_2O_2 (500 μM) and FA-97 (0, 0.25, 0.5, 1 μM) for 24 h. The relative expressions of HO-1, NQO-1 and Nrf2 were represented by densitometric analysis. Results are representative of three independent experiments and expressed as means ± SD. $^{\#\#}P$ < 0.01 compared with control group, $^{**}P$ < 0.01 compared with H_2O_2-stimulated group.

Figure S6.

Figure S6 shows the expression of HO-1, NQO-1 and Nrf2 in PC12 cells. This result indicated the total expression of HO-1 and NQO-1 was promoted by FA-97, while FA-97 had no effect on the Nrf2 expression. The histogram is related to Figure 4 and described in the results section (3.4) on page 13 line 28-30 of the manuscript.

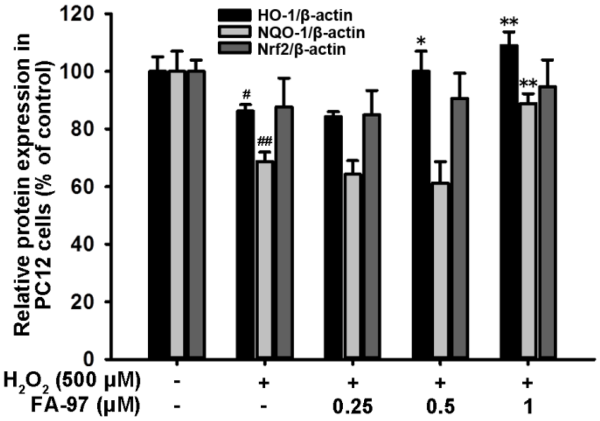

Figure S6.The effect of FA-97 on the expression of HO-1, NQO-1 and Nrf2 in PC12 cells. Cells were treated with H_2O_2 (500 μM) and FA-97 (0, 0.25, 0.5, 1 μM) for 24 h. The relative expressions of HO-1, NQO-1 and Nrf2 were represented by densitometric analysis. Results are representative of three independent experiments and expressed as means ± SD. $^{\#}P$ < 0.05, $^{\#\#}P$ < 0.01 compared with control group, $^{**}P$ < 0.01 compared with H_2O_2-stimulated group.

Figure S7.

Figure S7 shows Nrf2 expression in cytoplasm and nuclear of SH-SY5Y and PC12 cells. This result indicated the nuclear Nrf2 level was increased, while the Nrf2 expression in cytoplasm was inhibited by FA-97. The histogram is related to Figure 4 and described in the results section (3.4) on page 14 line 4-6 of the manuscript.

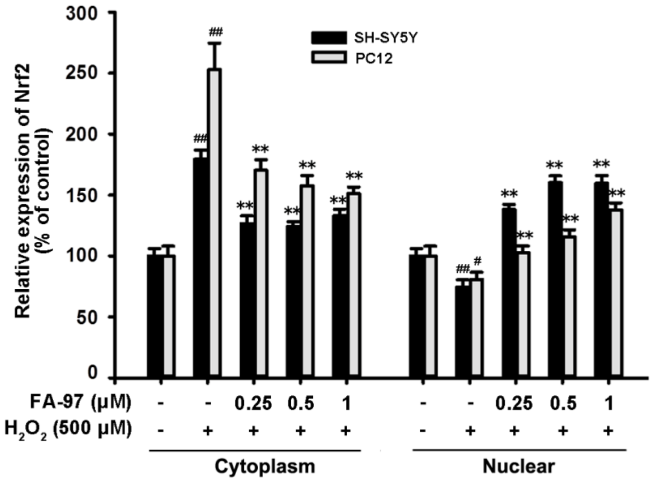

Figure S7.The effect of FA-97 on Nrf2 expression in SH-SY5Y and PC12 cells treated with H_2O_2 (500 μM) and FA-97 (0, 0.25, 0.5, 1 μM) for 24 h. The relative expression of Nrf2 in cytoplasm and nuclear was represented by densitometric analysis. Results are representative of three independent experiments and expressed as means ± SD. $^{\#}P < 0.05$, $^{\#\#}P < 0.01$ compared with control group, $^{**}P < 0.01$ compared with H_2O_2-stimulated group.

Figure S8.

Figure S8 shows the Bcl-2/Bax ratio in hippocampus and cortex of mice in each group. This result indicated FA-97 increased the Bcl-2/Bax ratio in SCOP-treated mice brain. The histogram is related to Figure 7 and described in the results section (3.7) on page 16 line 8-10 of the manuscript.

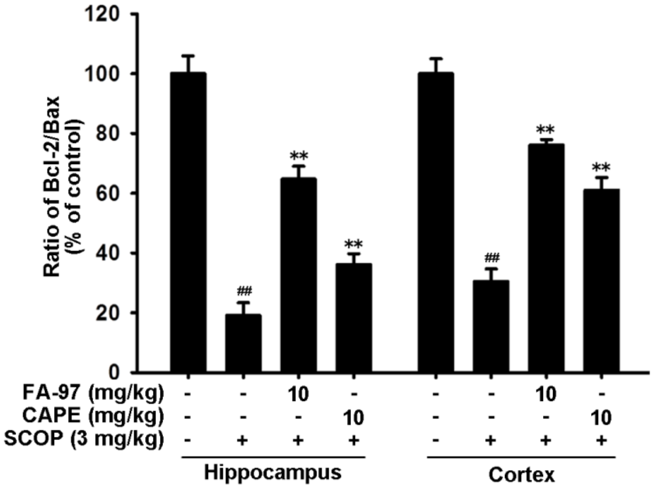

Figure S8. The effect of FA-97 on the expression of Bax and Bcl-2 in hippocampus and cortex of mice in each group. The Bcl-2/Bax ratio was represented by densitometric analysis. Results are representative of three independent experiments and expressed as means ± SD. $^{\#\#}P < 0.01$ compared with control group, $^{**}P < 0.01$ compared with SCOP-treated group.

Figure S9.

Figure S9. shows the Cytochrome C expression in hippocampus and cortex of mice in each group. This result indicated FA-97 reduced the amount of Cytochrome C in SCOP-treated mice brain. The histogram is related to Figure 7 and described in the results section (3.7) on page 16 line 10-11 of the manuscript.

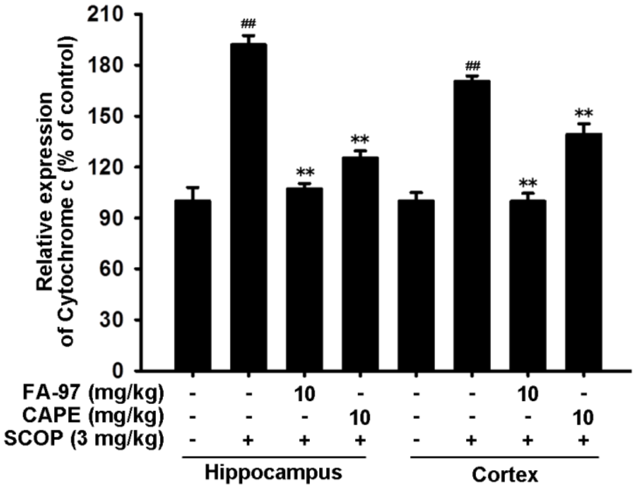

Figure S8. The effect of FA-97 on Cytochrome C expression in hippocampus and cortex of mice in each group. Relative expression of Cytochrome C was represented by densitometric analysis. Results are representative of three independent experiments and expressed as means ± SD. $^{\#\#}P$ < 0.01 compared with control group, $^{**}P$ < 0.01 compared with SCOP-treated group.

Figure S10.

Figure S10 shows the chemical structures of caffeic acid 4-*O*-glucoside, caffeic acid phenethyl ester (CAPE) and caffeic acid phenethyl ester 4-*O*-glucoside (FA-97). This is used to compare differences among three compounds and help describe the synthetic method of FA-97, by which the two different functional groups of CAPE and caffeic acid 4-*O*-glucoside can be connected with a linker to form a newly compound, in the light of pharmacophore combination principle in medicinal chemistry. All of these have been discussed in the second paragraph of discussion section on page 17 of the manuscript.

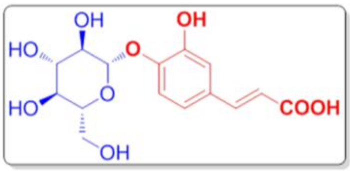

caffeic acid 4-*O*-glucoside

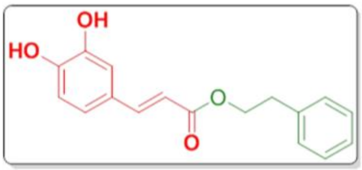

caffeic acid phenethyl ester (CAPE)

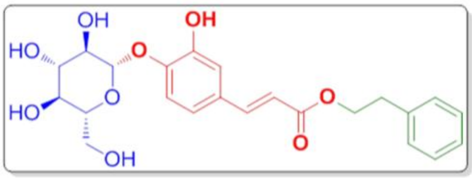

caffeic acid phenethyl ester 4-*O*-glucoside (FA-97)

Fig. S10. Chemical structures of caffeic acid 4-*O*-glucoside, caffeic acid phenethyl ester (CAPE) and caffeic acid phenethyl ester 4-*O*-glucoside (FA-97).
